# Effect of Post-Activation Performance Enhancement in Combat Sports: A Systematic Review and Meta-Analysis—Part I: General Performance Indicators

**DOI:** 10.3390/jfmk10010088

**Published:** 2025-03-09

**Authors:** Artur Terbalyan, Karol Skotniczny, Michał Krzysztofik, Jakub Chycki, Vadim Kasparov, Robert Roczniok

**Affiliations:** 1 Institute of Sport Sciences, Jerzy Kukuczka Academy of Physical Education, 40-065 Katowice, Poland; a.terbalyan@awf.katowice.pl (A.T.); k.skotniczny@awf.katowice.pl (K.S.); j.chycki@awf.katowice.pl (J.C.); kaspy996@gmail.com (V.K.); r.roczniok@awf.katowice.pl (R.R.); 2 Department of Sport Games, Faculty of Physical Education and Sport, Charles University in Prague, 162 52 Prague, Czech Republic

**Keywords:** countermovement jump, performance testing, strength training, plyometrics, martial arts

## Abstract

**Background/Objectives:** Post-activation performance enhancement (PAPE) has been explored for its potential to improve general performance in combat sports. This systematic review and meta-analysis investigated the effects of PAPE protocols on physical performance, focusing on differences across disciplines, competitive levels, and testing methods. **Methods:** A PRISMA-guided search (2010–2023) identified 19 studies examining PAPE protocols in combat sports athletes. The inclusion criteria required human trials using defined PAPE protocols, with outcomes of general performance indicators such as countermovement jumps (CMJs). A meta-analysis was conducted on data from 866 athletes using random effects modeling. **Results:** The PAPE protocols yielded a pooled effect size of 0.136 (95% CI, 0.008–0.263) across 866 athletes. Taekwondo athletes exhibited the most pronounced improvements in CMJ performance, particularly when using protocols that combined repeated vertical jumps with heavy-resistance cluster sets, and with dynamic, sport-specific movements such as the bandal chagui protocol achieving an effect size of 1.19 (*p* < 0.001). Conversely, Muay Thai athletes experienced performance declines when the protocols incorporated highly specific techniques, such as roundhouse kicks (ES = −1.36, *p* = 0.009). Analysis by competitive level revealed pooled effect sizes of 0.14 (95% CI, −0.01 to 0.29) for amateur athletes and 0.13 (95% CI, −0.11 to 0.38) for elite athletes, with no statistically significant differences observed between these groups. **Conclusions:** PAPE’s effectiveness depends on tailoring protocols to the competitive level and discipline. Short rest intervals support plyometric protocols for amateurs, while heavy-resistance exercises enhance elite performers. Further research is needed to standardize PAPE protocols and explore discipline-specific adaptations.

## 1. Introduction

Understanding the physiological determinants of performance is essential for optimizing training strategies and enhancing competitive outcomes [1,2,3]. The key features in athletic parameters of muscle strength and power are associated with improved force–time properties (such as rate of force development [RFD] and external mechanical power) and general sport skill performance (such as jumping, sprinting, and direction change) and lower injury rates [4]. An athlete’s overall performance is influenced by enhanced force–time characteristics, which are significantly correlated with greater muscular strength [4].

Based on its recent contractile experience, skeletal muscle may exhibit diminished or improved contractile capability. Through post-activation potentiation (PAP), certain contractions may cause an increase in muscular force, RFD, or both in subsequent contractions [5]. While phosphorylation of the myosin regulatory light chain (MRLC) is widely recognized as the primary mechanism driving the PAP effect, much of the research in this field has focused on performance-based outcomes rather than direct physiological markers of potentiation [6,7]. Moreover, while PAP is significant for only a few minutes [8], the peak voluntary performance enhancement often occurs 6–10 min after the conditioning activity (CA). Due to this discrepancy, the alternative term post-activation performance enhancement (PAPE) has been proposed, along with additional mechanisms that may contribute to acute performance improvements [6,9]. These include an increase in muscle temperature to enhance contractile efficiency and changes in muscle water content influencing tissue elasticity and stiffness [6]. Additionally, enhanced neural activation, reflected in improved motor unit recruitment and firing rates, further augments performance outcomes [6]. In training practice, this acute improvement in strength and power performance is typically induced through recent voluntary contractile activity, such as performing a maximum voluntary isometric contraction (MVIC) or another maximal effort exercise as a CA to enhance the subsequent athletic task [5].

The nature of combat sports presupposes a high level of power manifestation [10], which could be achieved through PAPE-based training protocols [11], obtaining the previously mentioned neuromuscular adaptations [5,6]. Power development is fundamental to performance in combat sports, where explosive strength underlies critical skills such as rapid kicks, punches, jumps, and quick changes of direction. In disciplines such as taekwondo, boxing, Muay Thai, judo, wrestling, and karate, effective power output can be the decisive factor between winning and losing. Recent research, including the work by da Silva Santos et al. [12], has emphasized the importance of assessing lower-body performance, particularly in taekwondo, where rapid force production is directly linked to effective kicking and dynamic movement. The PAPE phenomenon is typically integrated into training via methods such as complex training [13] and contrast training [14]. While both techniques are widely endorsed for enhancing athletic performance [15] and have gained considerable acceptance in the strength and conditioning community [16], they differ in their implementation and the specific neuromuscular activation strategies they employ.

A multitude of discriminant factors exist between individual combat sports athletes, including competitive level and discipline [17]. Therefore, the optimization of PAPE protocols for combat sports athletes, with an emphasis on adapting to these factors, should be studied to allow coaches to achieve maximal outcomes during the preparation process. To the best of the authors’ knowledge, there have yet to be published comprehensive studies reviewing the effect of CAs on the PAPE of combat sports athletes. To date, only one study has investigated the effectiveness of plyometric training interventions on enhancing the physical performance of combat sports athletes [18]. Hence, there is a need for research to evaluate the efficacy of various PAPE protocols, which may help to select the most appropriate training variables to elicit the PAPE effect. Additionally, the type of discipline, experience, fitness level, and competitive level, as well as the type of physical test, must be considered to solve the research problem. Therefore, this systematic review and meta-analysis aimed to determine the following:

(a) Are there CA protocols capable of eliciting PAPE in combat sports athletes?
(b) How do different combat sports disciplines respond to PAPE protocols?
(c) What type of test had the greatest PAPE effect?
(d) Does the level of competition affect the magnitude of the enhancement effect of CA?

## 2. Materials and Methods

### 2.1. Search Strategy

This study followed the PRISMA guidelines for a systematic literature review and was not registered in a public database. [19,20]. PRISMA stands for “Preferred Reporting Items for Systematic Reviews and Meta-Analyses”. We collected data through the following stages: literature verification, literature selection, selection criteria review, and final selection [21]. To identify appropriate studies for the current review, literature searches of the PubMed/Medline, SPORTDiscus and Web of Science, Scopus, EBSCO, and Google Scholar databases were conducted by a member of the research team. The literature search included published records from databases between January 2010 and May 2023. The following keywords were used as search terms: “combat sport*” OR “martial art*” OR “MMA” OR “kickbox*” OR “box*” OR “wrestl*” OR “judo” OR “taekwondo” OR “karate” OR “muay thai” OR “grappl*” OR “jiu-jitsu”) AND “post-warm-up” OR “pre-activity” OR “post-activation potentiation” OR “post-activation performance enhancement”. “*” indicates truncations to avoid missing data (e.g., kickboxING vs kickboxER).

### 2.2. Inclusion and Exclusion Criteria

Review papers, unpublished abstracts, theses, and dissertations were not included; only peer-reviewed, original research articles published in English and providing full-text accessibility were considered. To be accepted for inclusion, studies needed to include human experimental trials that examined healthy combat sports athletes actively participating in recognized disciplines (e.g., boxing, judo, karate, taekwondo, Brazilian jiu-jitsu, mixed martial arts, wrestling, kickboxing, Muay Thai). The participants were required to have no current injuries or known health conditions that might affect their neuromuscular performance. Crucially, eligible studies had to employ well-defined PAP or PAPE protocols, compare these to appropriate control conditions (e.g., rest, standard warm-up, or alternative intervention), and report changes in performance measures. The PICOS framework [20] was developed following the PRISMA guidelines to ensure systematic and standardized inclusion criteria (Table 1).

### 2.3. Text Screening

Titles and abstracts of the initial search results were independently assessed for relevance by two investigators (AT and KS), based on a priori inclusion and exclusion criteria. The same two researchers independently screened the full texts after screening the titles and abstracts to assess compliance with the inclusion and exclusion criteria, to further identify studies for inclusion in the analysis. Any disagreements among reviewers were discussed until a consensus decision was reached.

### 2.4. Data Extraction, Study Coding

The extracted information included the study authors, year of publication, study design, age, sex, competitive level, 1RM values in a range of strength measures, the combat sports discipline, CAs with training variables, and outcomes. The first and second authors gathered the data through the blind method. The authors collected the means with SD of pre- and post-conditioning performance test results. The third author was responsible for the initial screening and selection process. Any missing data were obtained by direct request to the corresponding author. In the absence of feedback, ImageJ© software (ImageJ v. 1.54d, National Institute of Health, Bethesda, MD, USA) was employed to obtain the requisite data. In cases where data were unavailable, the study was excluded. Effect sizes were calculated from the means and standard deviations of the pre-and post-intervention data. The results of the random effects meta-analysis are presented in a forest plot. The minimum number of studies included in the meta-analysis was five. All studies meeting the inclusion criteria were carefully reviewed to document relevant study characteristics and were tabulated in an Excel spreadsheet (Microsoft Corporation, Redmond, WA, USA).

### 2.5. Quality Assessment

The Physiotherapy Evidence Database (PEDro) scale [22] was employed by the initial and secondary authors of the review (AT and KS) to assess the risk of bias and the level of research quality of each study independently. In the event of any discrepancies between the two assessors, a consensus meeting was held to resolve them. Studies with a low or high risk of bias and studies with high research quality were given a score of 0 or 1 for each item. In the absence of clear information or evidence to the contrary, the item was assigned a score of 0. Studies were classified as excellent (PEDro score of 9 to 10), good (6 to 8), fair (4 to 5), or poor (0 to 3) according to their PEDro score. Studies with a PEDro score greater than 4 were included in the systematic review, while for the meta-analysis only studies scoring 6 or above were retained.

### 2.6. Meta-Analysis

The statistical analysis was conducted on meta-analyses of means, standard deviations, and effect sizes (ESs) computed using Hedges’ g as described by Hedges and Olkin [23]. Given that many of the selected studies had small sample sizes, this version of the formula was used to correct for potential bias in effect size estimates. Between-study heterogeneity was explored using forest plots and was evaluated statistically using *I*^2^, which represents the percentage of between-study variation that is due to heterogeneity rather than chance. A *I*^2^ of 0% indicates the absence of heterogeneity, while values of 50% or above suggest considerable heterogeneity. A random effects model was used since it is more conservative, and the observed heterogeneity in a few cases was 50%. Statistical analysis was carried out using PQStat Software (1.8.2.208, PQStat, Poznań, Poland) and data was visualized using PRISM 10 software (v. 10.1.1, GraphPad, MA, USA). The use of either funnel plots or formal statistical tests to explore publication bias was not employed, as the number of articles utilized for analysis was insufficient for the interpretation of funnel plots. Furthermore, the use of funnel plots can be misleading for the exploration of publication bias, particularly when the number of studies is relatively limited [24]. In addition to these meta-analytic techniques, a one-way analysis of variance (ANOVA) was conducted to compare effect sizes across different subgroups, such as combat sports disciplines and competitive levels. The significance level was set at *p* < 0.05.

## 3. Results

### 3.1. Literature Search

**Figure 1:**
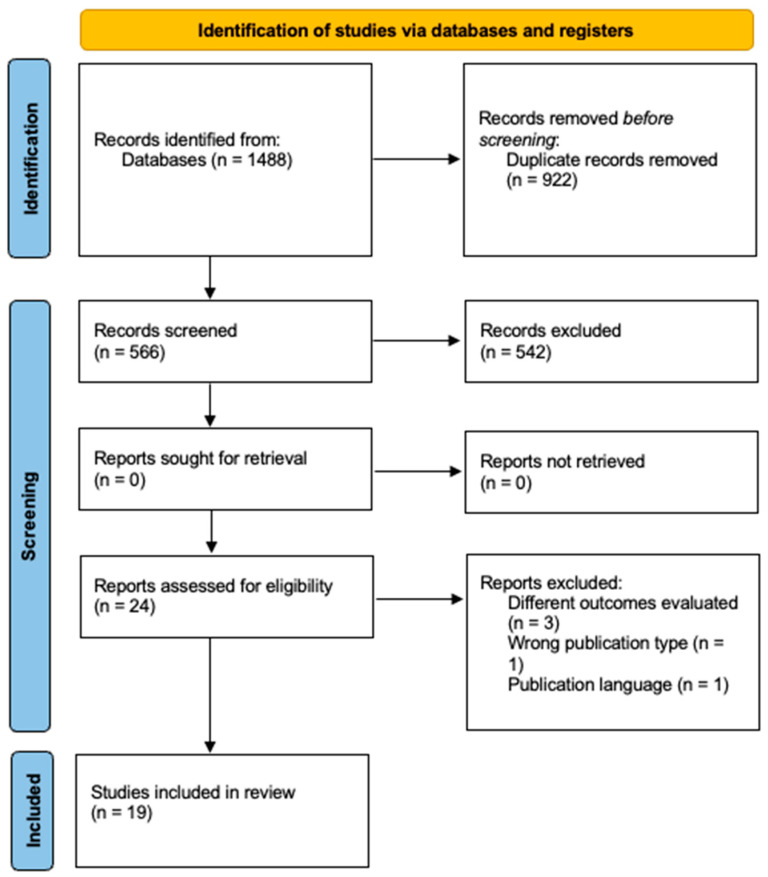
PRISMA chart of the search and study inclusion process.

Figure 1 illustrates the screening process, which was organized into three consecutive phases: (i) identification, (ii) screening, and (iii) inclusion in the review. Nineteen articles were ultimately selected to investigate PAPE in combat sports. Among these, eight articles focused on taekwondo [12,25,26,27,28,29,30,31], two on boxing [32,33], one on wrestling [34], one on judo [35], one on mixed martial arts [36], one on karate [37], one on kickboxing [38], one on Muay Thai [39], one on wushu [40], and two included mixed combat sports [41,42].

### 3.2. Quality Assessment

The mean PEDro score across all 19 studies was 7.8. Of these studies, 3 were classified as excellent (16%), 13 as good (68%), and 2 as fair (11%). No studies were classified as poor (0%). The results of the quality assessment are presented below in Table 2.

### 3.3. Systematic Review

The 19 studies included in this systematic review (Table 3) investigated PAPE interventions across combat sports, including taekwondo (*n* = 8), wrestling (*n* = 1), judo (*n* = 1), boxing (*n* = 2), and a mixed group of participants (*n* = 2). The remaining studies investigated mixed martial arts (MMA; *n* = 1), karate (*n* = 1), kickboxing (*n* = 1), Muay Thai (*n* = 1), and taolo wushu (*n* = 1). Female athletes were included in 2 studies, males in 12, while 5 studies included mixed-gender groups. The sample sizes ranged from 8 to 48 participants. The aggregate sample size across the 19 studies included in the systematic review was 286 participants. The majority of participants were amateurs (*n* = 13 studies), while 4 studies focused on elite athletes and 2 on mixed athlete status. The majority of studies target participants aged 16–25 years, with some including broader age ranges up to 29 years. The outcome measures included general performance metrics. The countermovement jump was the most frequently assessed parameter (*n* = 12 studies), followed by isokinetic strength, running anaerobic sprint test, hip power test, bench press throw, and dumbbell press tests. The study designs included randomized controlled trials (RCTs) (*n* = 12), crossover trials (*n* = 5), and quasi-experimental methods (*n* = 2). All the studies employed a pre- and post-intervention comparison to evaluate the PAPE effect. The CAs employed were diverse, encompassing loaded squat jumps, heavy-resistance training (e.g., squats, bench presses), plyometric drills (e.g., tuck jumps, broad jumps), ballistic push-ups, isokinetic exercises, elastic resistance punches, and sport-specific protocols such as roundhouse kicks and other techniques like whole-body vibration.

### 3.4. Meta-Analysis

Nine studies provided sufficient data for inclusion in the meta-analysis. These studies reported the requisite statistical information—such as means and standard deviations—for CMJ performance, which was selected as the primary outcome measure for the meta-analysis due to the limited availability of comprehensive data for the other performance indicators. The meta-analysis was performed on the effects of selected CAs extracted from each study. The data synthesis process resulted in the inclusion of 62 CAs in the meta-analysis input (Table 4).

**Figure 2:**
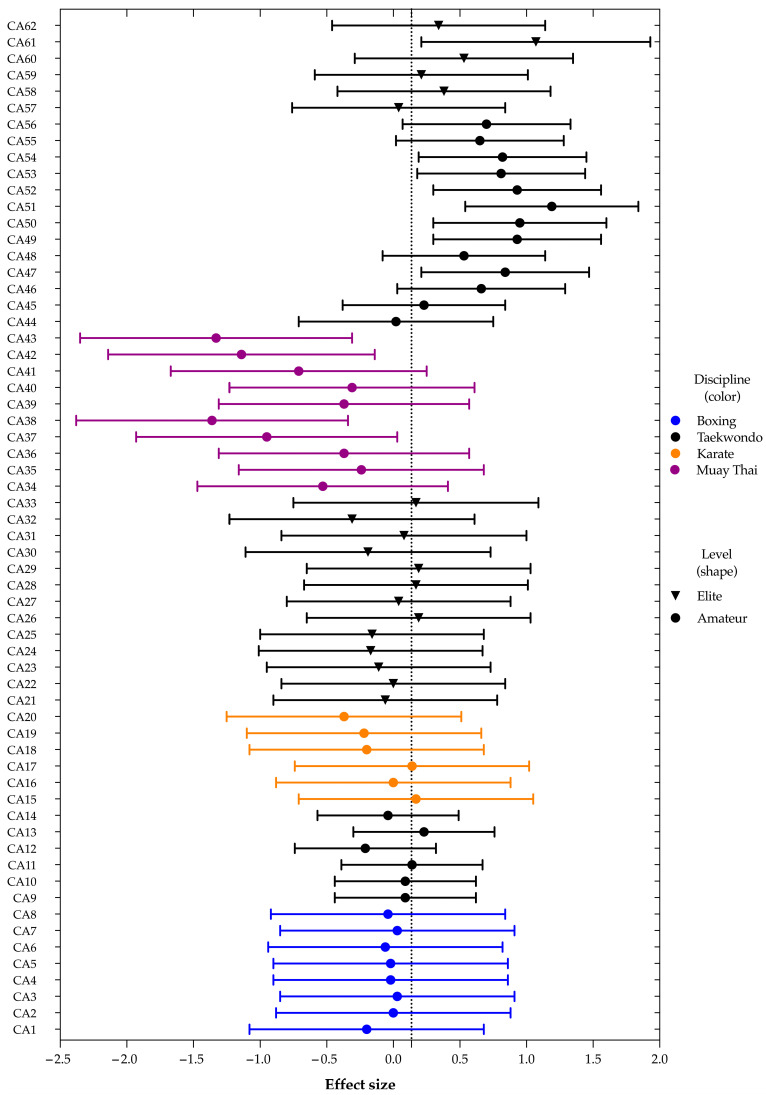
Forest plot for general outcomes. Random effects model showing Hedges’ effect for each of the 62 included cases from the 9 studies and pooled for all. Pooled mean for Hedges’ effect 0.136 (95% CI, 0.008–0.263); *I*^2^ = % (40.81%; CI 95% 19.61–56.43%). Size boxes are proportional to study sample size.

Significant heterogeneity was found among the included studies, with an *I*^2^ greater than 40% for the outcomes analyzed, hence the decision to use a random effects model. A meta-analysis of the ES was performed, and forest plots were used to describe the ES. Individual results for each of the 62 cases from the nine studies included in the meta-analysis are presented in terms of ES and 95%CI, together with pooled results for all the studies, where the size of the boxes is proportional to the study sample size. The total sample size is 866. The pooled mean for the ES was 0.136 (95% CI, 0.008–0.263) (Figure 2, Table 5). It was found that in the 25 CAs, the mean for the ES was higher than the pooled mean of the ES, especially for Oergui et al. [30] and Boyaci and Kizilet [31], all of them for taekwondo. It was found that in the 35 CAs, the mean for the ES was lower than the pooled mean of the ES, especially for Cimadoro, et al. [39].

**Figure 3:**
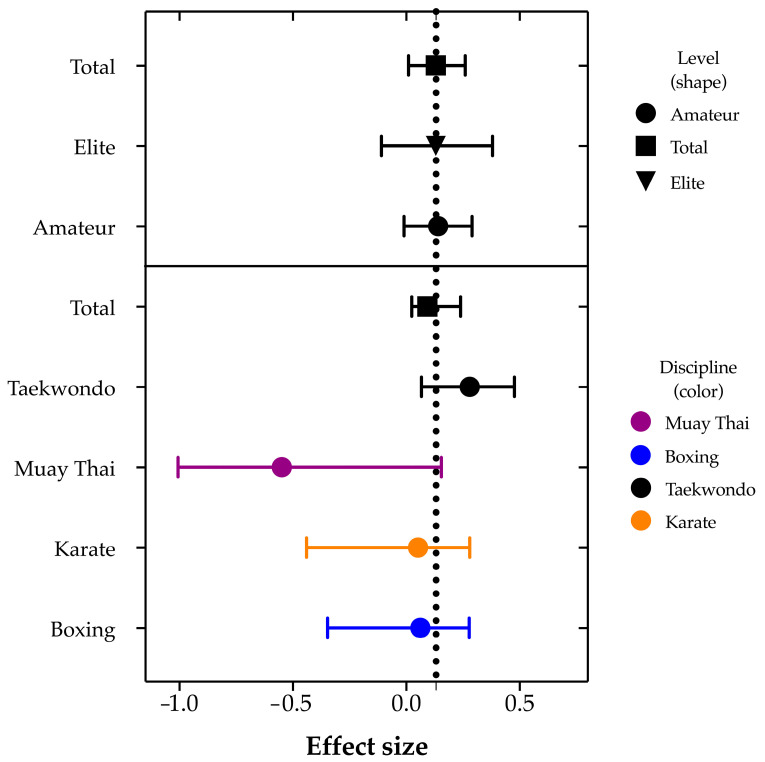
Forest plot (random effects model) showing aggregated Hedges’ effect sizes by discipline and competitive level for each of the 62 included cases from 9 studies, as well as the pooled estimate for all cases. The pooled mean Hedges’ effect sizes were 0.131 (95% CI, −0.044–0.280) and 0.13 (95% CI, 0.01–0.25), respectively. The size of the squares is proportional to the study sample size. Nine studies, as well as the pooled estimate for all cases.

The results of the meta-analysis conducted for each discipline confirmed the findings previously obtained for the entire sample. The pooled mean Hedges’ effect size was 0.131 (95% CI, −0.044–0.280) (Figure 3, Table 6). In 11 reports, the mean ES exceeded the pooled mean, most notably in the studies by Oergui et al. [30] and Boyaci and Kizilet [31], both of which examined taekwondo. Conversely, the mean ES was lower than the pooled mean in three reports, most notably in the study by Cimadoro et al. [39], which focused on roundhouse kicks (20 × 1). The analysis further showed that for Muay Thai, the Hedges’ effect was significantly lower at −0.70 (95% CI, −1.01–−0.397) compared to the overall mean Hedges’ effect of 0.131 (95% CI, 0.024–0.239). In addition, an ANOVA was conducted for each discipline and across groups. The results confirmed significant differences between disciplines (F = 44.61; df = 3; *p* < 0.0001) and overall (F = 102.55; df = 61; *p* = 0.001).

The results of the meta-analysis by competitive level confirmed the findings previously obtained for the entire sample. The pooled mean of the ES was 0.13 (95% CI, 0.01–0.26) (Figure 3, Table 7). For 11 reports, the mean Hedges’ effect size exceeded the pooled mean, particularly in the studies by Oergui et al. [30] and Boyaci and Kizilet [31], which focused on taekwondo (10 amateur and 1 elite). Conversely, in three reports, the mean Hedges’ effect size was lower than the pooled mean, notably in the study by Cimadoro et al. [39] examining roundhouse kicks (20 × 1) at the amateur level. Additionally, an ANOVA was performed by competitive level, as well as within and between groups. This analysis did not reveal any statistically significant differences (*p* > 0.05).

## 4. Discussion

After a CA, the potentiation effect is ambiguous and dependent on variables related to training experience, the potentiation procedure, and the practiced combat sports discipline. Despite numerous studies, a precise consensus regarding the optimal acute conditioning mode protocol for recreationally trained, trained, and athletic populations has yet to be formed. To address this knowledge gap, we conducted a meta-analysis of nine studies (62 CAs) to quantitatively identify which conditioning protocol components (type of exercise, intensity, and volume) optimize general performance indicators in combat sports athletes, and how these variables are affected by competitive status and combat sports discipline.

This study specifically addressed key questions central to power development in combat sports: (a) Which CA protocols effectively elicit PAPE in athletes? (b) How do various combat disciplines—including taekwondo, boxing, Muay Thai, judo, wrestling, and karate—respond to these protocols? (c) What is the most sensitive performance test for capturing the PAPE effect? (d) Does the competitive level modulate the magnitude of the PAPE response? The present findings underscore that dynamic, sport-specific protocols—exemplified by the bandal chagui method in taekwondo—consistently yield substantial improvements in explosive performance, as measured by the CMJ. In contrast, certain protocols in disciplines like Muay Thai may even reduce performance, highlighting the critical importance of aligning CAs with the neuromuscular and biomechanical demands specific to each combat sport. Moreover, while both amateur and elite athletes exhibit PAPE responses, the variability observed suggests that competitive level and training status must be carefully considered when designing and implementing these interventions.

In addressing the first research question, our meta-analysis revealed that PAPE protocols, overall, produced a pooled effect size of 0.136 (95% CI, 0.008–0.263). Notably, protocols that combined dynamic actions—such as repeated vertical jumps with adequate recovery (3–7 min of rest)—with heavy-resistance cluster sets (performed at 75% 1RM with optimized intra- and inter-set rests) were particularly effective. These dynamic protocols are likely more effective because they closely mimic the explosive movements inherent in combat sports.

Regarding discipline-specific responses, the data show that taekwondo athletes experienced the most pronounced enhancements in CMJ performance. This is likely due to the sport’s emphasis on rapid, explosive lower-body actions, as supported by the work of da Silva Santos et al. [12]. Conversely, in Muay Thai, highly specific CAs—such as roundhouse kicks—appeared to be detrimental to performance, suggesting that not all sport-specific techniques translate into a positive PAPE response.

When considering the sensitivity of performance tests, the CMJ emerged as the most frequently employed and reliable measure across studies. Its responsiveness to changes in explosive power makes it an ideal tool for assessing the PAPE effect in combat sports, where rapid force production is paramount.

Finally, analysis by competitive level revealed pooled effect sizes of 0.14 (95% CI, −0.01 to 0.29) for amateur athletes and 0.13 (95% CI, −0.11 to 0.38) for elite athletes, with no statistically significant differences between these groups. This finding suggests that while competitive status influences PAPE outcomes, the design of the conditioning protocol—particularly in relation to rest intervals and exercise specificity—plays a critical role in determining the magnitude of performance enhancement.

### 4.1. Main Findings

The primary findings of this study were that not all types of conditioning were effective in enhancing general performance variables. The most considerable body of scientific data pertains to the use of CA in the context of performance enhancement, as measured by the CMJ. For example, Boyaci and Kizilet [31] demonstrated that utilizing a cluster set (three sets) in the barbell squat at a 75% 1RM load with a 4 min rest between sets and a 3 min interval within the series yielded the most pronounced enhancement in distance in the jump test relative to the conventional sets type. In comparison, in a study by Ouergui et al. [30], improvements in general performance were seen after a dynamic type of potentiation. Participants performed three sets of repeated vertical jumps (5 s of consecutive jumps). The largest PAPE effect was observed with a 45 s intra-set and self-selected intra-set rest with a 3 min rest between sets.

In contrast, Ferreira et al. [41] found no additional effect on mean and peak power when performing a power bench press after completing six reps at 50% 1RM in the same exercise. It appears that one set may not be sufficient to achieve the PAPE effect. In another study, five sets of bench presses improved performance in bench press throw on 30% 1RM [42]. A positive effect was noticed after five sets on 65% 1RM with a 4 min rest, as well as after five sets on 85% 1RM with both a 4 and 8 min rest between sets. However, these studies were not included in the meta-analysis due to the different type of test assessing general performance and insufficient evidence for inclusion. Different outcomes with various strategies emphasize the importance of analyzing the PAPE methodology, especially since a variety of strength-training concepts for athletes are widely recognized in the literature [43]. The importance of defining appropriate training modalities for each combat sport cannot be overemphasized, as this aspect of the sport is often neglected, leading to suboptimal results [44].

Nevertheless, the greatest positive effect was observed for the specific type of potency that is bandal chagui, in the form of three sets with a 7 min rest between sets and a 30 s intra-set rest. It is also worth noting that the specific form of potentiation associated with one series of 20 roundhouse kicks with a 30 min rest and 3 s intra-set rest resulted in a deterioration in general performance (ES = 1.36; *p* = 0.009) [39]. The findings suggest the necessity for the examination of efficacious potentiation techniques, specifically in the context of resistance training, across a range of high-intensity and dynamic activities, such as repeated jumps under conditions where no positive effect of potentiation was observed.

Overall, the results of our meta-analysis indicate that dynamic, sport-specific conditioning protocols produce superior PAPE effects compared to traditional heavy-resistance approaches. In particular, the bandal chagui protocol, exemplified by CA51—which involves three 5 s bouts with 7 min rest intervals and a 30 s intraset recovery in taekwondo athletes—produced an effect size of 1.19 (*p* < 0.001). This finding suggests that such dynamic protocols, which closely mimic the explosive movement patterns inherent to martial arts, may be more effective in priming the neuromuscular system. Other dynamic protocols, such as those involving consecutive vertical jumps (CA54-CA56), showed effect sizes ranging from 0.82 to 0.95, further supporting the efficacy of sport-specific conditioning in producing pronounced performance improvements. In contrast, heavy-resistance protocols—such as those examined by Boyaci and Kizilet [31] (CA57-CA62)—achieved slightly lower effect sizes; for example, CA61, a squat-based cluster set performed at 75% 1RM with a 4 min rest between cluster sets and a 180 s recovery between sets, yielded an effect size of 1.07.

In conclusion, although both dynamic and high-resistance CAs can facilitate PAPE, our results suggest that dynamic protocols—particularly the bandal chagui method—may provide a slightly superior stimulus for improving explosive performance in kicking martial arts.

### 4.2. The Competitive Level Factor

The meta-analysis of the data relating to the effect of the competitive level factor indicated inconclusive results. In the context of amateur athletes, the influence of potentiation can lead to either enhanced or diminished general performance. A reduction in athletic performance as measured by CMJ was noted in amateur Muay Thai athletes as a result of performing a specific form of warm-up [39]. A possible factor in the changes obtained could be the high volume of technical–tactical training associated with the execution of movement sequences similar to the exercise used as a form of potentiation. Perhaps the effectiveness of a CA is closely linked to its specificity to the performance measure being assessed; aligning the CA with the particular neuromuscular and biomechanical demands of the sport may optimize the PAPE effect [16,45]. Although achieving such a specificity may not always be feasible, this consideration underscores the importance of tailoring PAPE protocols to the unique performance requirements inherent to combat sports, thereby reinforcing their relevance as outlined earlier. In a study by Oergui et al. [30], the results demonstrated that the performance in all tests exhibited a notable improvement following the implementation of conditioning in amateur taekwondo athletes when compared to the control condition. It can be observed that plyometric exercises tend to favor a rest period of approximately three minutes, regardless of the effort-to-pause ratio employed. Conversely, repeated high-intensity techniques appear to favor a longer rest period, of around seven minutes, particularly when ratios of 1:6 and 1:9 are utilized. The superiority of longer intervals in inducing the PAPE effect could be a result of positive balance between fatigue and neuromuscular potentiation and the intensity of the load used [46]. This finding is consistent with the aforementioned results. For elite combat sports athletes, it appears that CA may improve general performance or show no effect at all. Heavy barbell squats were found to have a positive influence on the height of the CMJ test in elite taekwondo athletes [31]. In summary, these findings suggest that the optimal PAPE response is highly contingent on both the athlete’s competitive level and the specific CA employed, with amateurs exhibiting more variable outcomes and elite athletes tending to respond more consistently to high-load protocols.

In addition, Langer et al. [36] showed that amateurs achieved greater force and RPD results after the ballistic push-up as an activation exercise performed at 50% 1RM, whereas force and RPD were greater at RFD at 65% 1RM. For elites, the highest force was achieved at 80% 1RM, power at 50% 1RM, and RFD and RPD at 80% 1RM. In comparison, Castro-Garrido et al. [28] indicated that the CMJ did not significantly differ between athletes nor between the intervention, competitive level, or conditions, which were a half-squat on 95%1RM with three sets of three reps, three sets of 10 jumps, or a combination of both exercises. In another study, an elastic resistance pull exercise combined with broad jumps resulted in greater peak power during the high-pull test compared to a protocol that focused solely on lower-body conditioning [35]. This finding reinforces the importance of aligning the CA with the specific performance measure. Since the high-pull test primarily engages the upper body, incorporating an upper body-focused CA appears to be more effective than simply increasing the total volume of activity or involving a greater number of muscle groups.

In the other studies included in the meta-analysis, no improvement in physical performance was observed in elite athletes. However, it is doubtful that this would lead to a deterioration in their athletic performance. The competitive level can therefore potentially influence the PAPE effect. Individuals with a lower level of experience can achieve significant improvement in physical performance but also a decline in performance. This may possibly be attributed to the balance between the mechanical properties of PAPE and the accumulation of fatigue resulting from high-intensity exercise [8]. For example, Wilson et al. [47] indicate that individuals with little training experience showed a decrease in power of approximately 120% when performing multiple sets compared to single sets. In contrast, trained individuals and experienced athletes increased their power by approximately 104% and 320%, respectively, when performing multiple versus single sets. Chronic resistance training improves fatigue resistance by increasing buffering capacity [48,49] and overall resistance to skeletal muscle injury [50]. These data suggest that when transitioning from single to multiple sets in less trained individuals, CA may cause significantly more fatigue than the PAPE effect can overcome. However, in our study, this cannot be unequivocally confirmed and is only a presumption.

In addition to the level of fitness itself, it is worth considering the potential impact of the personal profile related to muscle fiber composition [51], which could potentially be correlated with fitness level [52]. The post-activation enhancement effect is greater in fast-twitch fibers [53], which have lower basal calcium ion sensitivity [54,55] and greater myosin light chain kinase activity than slow-twitch fibers [53]. This, however, remains in the realm of speculation ,as the studies included did not verify muscle fiber composition. Attempting to estimate muscle fiber composition may be a suggestion for future research projects that may try to directly characterize athletes via muscle biopsy [56,57] or indirectly, for example via tensiomyography [58,59].

### 4.3. The Discipline Factor

The above considerations should also include the impact of the combat sports discipline on susceptibility to the PAPE effect. The performance enhancement effect was observed in taekwondo athletes when sports discipline was taken as a differentiating factor, whereas deterioration in physical performance was noted in Muay Thai athletes. In the case of boxing and karate, the effect related to the influence of potentiation was not statistically significant, regardless of whether this referred to an improvement or worsening of general performance. Nevertheless, it would be prudent to maintain skepticism in ascribing the influence of the specific discipline as a determinant of the PAPE effect. In fact, the data on Muay Thai fighters are derived from a study where the potentiation exercises were related to the execution of roundhouse kicks. Taekwondo was the most studied discipline with the highest number of CAs. The result may therefore be more an effect of the type of CA rather than the type of sport. The authors suggest adapting taekwondo’s CAs to other combat sports disciplines to make more consistent conclusions. Furthermore, the participants were amateurs, which, when considered alongside the nature of the activity, may have exerted an influence on the outcomes observed. It is possible that a different form of exercise, for instance, one that incorporates high-intensity resistance training, might have yielded entirely disparate results. The PAPE effect has been observed in taekwondo athletes; however, the majority of the results have been reported by Oergui et al. [30] with the bandal chagui and consecutive vertical jump protocols previously discussed. It would appear that greater consideration should be given to the type of exercise undertaken and the level of training experience, rather than the specific sport, when examining the PAPE effect.

Reliably evoking the PAPE response arises from a careful balance between the athlete’s training experience, their achieved strength level, the intensity of the CA, and the duration of the subsequent rest period. Crucially, the specific combat sport discipline also plays a pivotal role in determining the effectiveness of PAPE protocols. In practice, substantial differences in jump height have been documented across various disciplines [60], with the magnitude of these differences ranging from negligible to exceptionally large. These variations highlight that the neuromuscular and biomechanical demands inherent to each sport can markedly influence the response to PAPE interventions. Consequently, it is essential to customize the CA to meet the unique demands of each discipline and to fine-tune the rest intervals accordingly, as deviations from the optimal duration may lead to fatigue accumulation and a diminished PAPE effect [5,47,61,62].

### 4.4. Limitations

It should be noted that this study is not without limitations. Firstly, the number of included studies was relatively limited (*n* = 9), which restricts the generalizability of the findings. The included studies primarily investigated a limited range of CAs, thereby leaving several potential variations (e.g., isometric potentiation, plyometric variations) underexplored. Secondly, the heterogeneity of methodologies across the studies (e.g., differences in testing protocols, rest intervals, and exercise selection) may have introduced variability, limiting the precision of the meta-analysis. Furthermore, discrepancies in competition status (amateur vs. elite), specific combat sports disciplines, and inconsistencies in participant characteristics (e.g., age, sex, and experience) may have introduced confounding variables. Moreover, the use of general performance measures, such as CMJ, may not fully represent the overall performance in combat sports, which is multifaceted. In addition, the observed results may have been influenced by variations in study design, particularly the use of small sample sizes and an inconsistent statistical power across the included studies.

It is noteworthy that many of the included studies employed relatively low loads in their CAs. This choice likely reflects a strategic attempt to balance the potentiation benefits with the risk of inducing excessive fatigue, which can negate the acute performance enhancements associated with PAPE. Lower loads may allow for sufficient neuromuscular activation while minimizing fatigue, particularly in protocols targeting explosive movements like the countermovement jump. However, this approach also raises questions about the potential trade-offs in maximizing muscle activation versus avoiding fatigue, especially when considering the different demands of various combat sports.

One potential limitation of our study is related to the participant group in Ferreira et al. [41]. Although the protocol used in this study is typical for team and combat sports, the subjects were not competitive athletes. Without further clarification from the original authors, it is not possible to fully verify that these participants meet our inclusion criteria for healthy, competitive athletes. Consequently, caution is warranted when interpreting the findings from this study in relation to competitive athlete populations.

### 4.5. Practical Applications

From a practical standpoint, the findings of this study offer insights for coaches and practitioners engaged in the training of combat sports athletes. CAs must be meticulously tailored to the athlete’s competition level and specific objectives. For amateur athletes, dynamic potentiation exercises such as repeated vertical jumps appear to be an effective method, provided that adequate rest intervals of approximately three minutes are observed. For elite athletes, the incorporation of heavy-resistance exercises, including barbell squats, may prove beneficial in enhancing performance, although it is essential to exercise caution in managing fatigue. It is of the utmost importance to optimize rest intervals, as plyometric exercises tend to favor shorter rest periods, while repeated high-intensity techniques require longer durations of recovery to achieve the desired PAPE effect.

The specific type of exercise is also a significant factor to be considered. Sport-specific dynamic activities, such as roundhouse kicks, have the potential to enhance performance. However, if the protocol is not properly designed, there is a risk of fatigue accumulation and a subsequent deterioration in performance. It is incumbent upon coaches to exercise discernment in the selection and testing of CAs, to identify those that are most suited to their athletes. While the results of this study do not definitively indicate discipline-specific effects, positive responses have been consistently observed in taekwondo athletes. It is recommended that coaches in other combat sports, such as Muay Thai, boxing, and karate, investigate conditioning protocols that integrate both resistance training and plyometric exercises.

### 4.6. Future Directions

It is recommended that future research addresses the limitations identified above by incorporating several key areas. Firstly, studies utilizing larger, more diverse cohorts are required to enhance their statistical power and improve the generalizability of their findings. The development of standardized protocols for potentiation procedures, including the implementation of consistent rest intervals, effort-to-pause ratios, and CA intensity, will assist in reducing the variability observed across studies. A further avenue for research would be to investigate the differences in PAPE effects across competition levels, namely novice, trained, and elite athletes. This would enable a deeper understanding of the interplay between training experience, fitness, and potentiation responses. 

Furthermore, combat sport-specific analyses should be expanded to explore the influence of multiple conditioning protocols on general indicators. Longitudinal studies examining the chronic effects of repeated potentiation sessions over extended training cycles will be crucial to determining their long-term impact on combat sports performance. To gain a comprehensive understanding of the effects of potentiation on combat sports performance, it is essential to incorporate a range of performance measures, including assessments of strength, power, reaction time, and endurance. This will enable researchers to capture the multidimensional nature of combat sports demands. Finally, future research should continue to investigate the balance between fatigue and potentiation, focusing on the physiological mechanisms that underlie the PAPE effect in various conditioning scenarios. Future research could also make an attempt to explore whether slightly higher loads—while carefully managing recovery—could further optimize the PAPE response without compromising performance, particularly in elite athletes whose training status might permit more aggressive loading strategies.

## Figures and Tables

**Table 1:** PICOS framework.

Factor	Description
Participants	Healthy combat sports athletes actively participating in recognized disciplines such as boxing, judo, karate, taekwondo, Brazilian jiu-jitsu, MMA, wrestling, kickboxing, and Muay Thai. Athletes must be free of injuries and any health conditions that could affect neuromuscular performance
Interventions	Studies must implement a defined post-activation performance enhancement (PAPE) protocol, specifying training parameters such as intensity (%1RM), volume (sets/reps), intra- and inter-set rest periods, and exercise type. Pre- and post-intervention performance metrics must be compared.
Comparisons	Control or comparison conditions, including rest, standard warm-up, alternative interventions, or between different PAPE protocols. Studies may also compare effects by discipline, competition level, or test results.
Outcomes	Acute changes in general performance metrics, including but not limited to countermovement jump (CMJ), isokinetic strength, sprint performance, power outputs, or agility tests. Results must be reported with means, standard deviations, or effect sizes.
Study design	Experimental designs such as randomized controlled trials, crossover designs, or quasi-experimental studies. Studies must include clearly defined protocols and outcome measures

**Table 2:** PEDro scale quality assessment.

#	Reference	1	2	3	4	5	6	7	8	9	10	11	Sum
1	Yi et al., 2022 [32]	yes	1	1	1	1	0	1	1	1	0	1	8
2	Eroglu et al., 2022 [34]	yes	0	1	1	0	0	1	1	1	1	1	7
3	Langer et al., 2022 [36]	yes	0	1	0	0	0	1	1	1	0	0	4
4	Ferreira et al., 2012 [41]	yes	1	1	1	1	0	1	1	1	0	0	7
5	Liossis et al., 2013 [42]	yes	1	1	1	0	0	1	1	1	1	1	8
6	Finlay et al., 2022 [33]	yes	1	1	1	1	0	1	1	1	1	1	9
7	Ouergui et al., 2022 [25]	yes	1	1	1	1	0	1	1	1	1	1	9
8	Margaritopoulos et al., 2015 [37]	yes	1	1	1	1	0	1	1	1	1	1	9
9	Da Silva Santos et al., 2015 [26]	yes	1	1	1	1	0	1	1	1	1	1	9
10	Pyun et al., 2022 [27]	yes	0	0	0	0	0	1	1	1	1	1	5
11	Afonso et al., 2021 [40]	yes	1	1	1	1	0	1	1	1	1	1	9
12	Catsro-Gatrido et al., 2020 [28]	yes	1	1	1	1	0	1	1	1	1	0	8
13	da Silva Santos et al., 2016 [12]	yes	1	1	1	1	0	1	1	1	1	1	9
14	Lum, 2019 [35]	yes	1	1	1	1	0	1	1	1	0	1	8
15	Cimadoro et al., 2018 [39]	yes	1	1	1	1	1	1	1	1	1	1	10
16	Oliviera et al., 2018 [29]	yes	1	0	1	1	0	1	1	1	0	1	7
17	Ouergui et al., 2023 [30]	yes	1	1	0	1	1	1	1	1	1	1	9
18	Boyaci & Kizilet, 2023 [31]	yes	1	0	1	0	0	0	1	1	1	1	6
19	Yilmaz et al., 2018 [38]	yes	1	1	1	1	1	1	1	1	1	1	10

The included studies were evaluated using the Physiotherapy Evidence Database (PEDro) scale, which classifies studies into four quality levels: excellent (scores of 9–10), good (6–8), fair (4–5), and poor (0–3).

**Table 3:** Characteristics of studies included in systematic review.

Ref.	AGE (M ± SD)	SEX	Level	1RM	D	*n*	CG	CA	L	RI, min	ISR, s	GPT
Yi et al., 2022 [32]	19–20 ± 1.55	M	A	90.80 ± 8.39 kg. squat	BOX	10	X	LSJ Squat	LSJ:4 × 8@30% BE SQ:3 × 5@80% HRE	3/6/9/12	90	CMJ
Eroglu et al., 2022 [34]	19.3 ± 1.2	M	A	86.3 ± 5.6 kg. squat	WRE	22	X	Squat	1 × 85%1RM	<1/3/6/9	X	CMJ
Langer et al., 2022 [36]	25 ± 7.3	M	MIX	X	MMA	48	NR	BPU/DBP	MIX	4	X	DBPT
Ferreira et al., 2012 [41]	25 ± 4	M	A	74 ± 8 kg. BP	MIX	11	X	BP	6x@50%1RM	NR	NR	PBPT
Liossis et al., 2013 [42]	26.1 ± 3.4	M	A	83.9 ± 8.4 kg. BP	MIX	9	NR	BP BPT	BP:5 × 65%/5 × 85%1RM BPT:3x@30%1RM	4	X	BPT
Finlay et al., 2022 [33]	19.7 ± 1.2	M	A	X	BOX	10	Y	ERP ISOP	ERP:2 × 5 ISOP:3 × 3s	3	NR	CMJ
Ouergui et al., 2022 [25]	16 ± 1	MIX	A	X	TKD	27	Y	RHIT CMJ	RHIT:3 × 5s CMJ:3 × 5s	<1	SSR	CMJ
Margaritopoulos et al., 2015 [37]	18.4 ± 1.2 (M); 19.2 ± 0.4 (F)	MIX	A	X	KAR	10	X	TJ	3 × 5	<1	30	CMJ
Da Silva Santos et al., 2015 [26]	20.3 ± 5.2	M	E	136.4 ± 30.7 kg. HS	TKD	11	Y	HS HS + J J	HS:3 × 1@95% J:3 × 10@40cm HS + J:3 × 2@95% + 4	5/10/SSR	3/30	CMJ
Pyun et al., 2022 [27]	20.2 ± 1.5	MIX	A	X	TKD	18	X	ISO EXT	%MV varied (10–40%, 40–10%)	NR	X	ISO
Afonso et al., 2021[40]	19.9 ± 3.3	F	E	53.90 ±27.2 kg. squat	TW	10	X	Squat	2 × 4@70%1RM	5 min	NR	RAST
Gastro-Gatrido et al., 2020 [28]	20.5 ± 2.38 (A); 24.75 ± 4.27 (E)	M	MIX	X	TKD	8	Y	HS HS + J J	HS:3 × 3@95% HS + J:3 × 2@95% + 4 J:3 × 10	<1/10	3/30	CMJ
da Silva Santos et al., 2016 [12]	20.3 ±5.2	M	E	132.8 ± 32.5 kg. HS	TKD	9	Y	HS	1 × 3(50%) 1 × 3(90%) 3 × 3(50%) 3 × 3(90%)	10	NR	CMJ
Lum et al., 2019 [35]	16–29	M	A	X	JU	11	Y	ER pull + BJ/BJ	2 × 5 or 3 × 5 BJ/ER pulls	3	X	HPT
Yilmaz et al., 2018 [38]	22.38 ± 4.01	M	A	X	KBOX	15	Y	HS	3 × 3@75%1RM	2	10	ISO
Cimadoro et al., 2018 [39]	20 ± 4	M	A	X	MT	9	NR	Kicks	20 × 1	<1	3	CMJ
Oliviera et al., 2018 [29]	18.6 ± 2.1	MIX	A	NR	TKD	15	X	WBV	1min@26Hz	NR	NR	CMJ
Ouergui et al., 2023 [30]	20.4 ± 1.4	MIX	A	X	TKD	21	Y	RHIT/P	RT:3 × 5s P:3 × 5s(40cm)	3/7/SSR	30/45/SSR	CMJ
Boyaci & Kizilet, 2023 [31]	15.17 ± 0.718	F	E	X	TKD	12	X	Squat, TS/CS	TS:3 × 12@75% CS:3 × (4 + 4 + 4)@75%	<1/4/8	180	CMJ

1RM—one maximal repetition test; A—amateur; BE—ballistic exercise; BJ—broad jump; BOX—boxing; BP—bench press; BPT—bench press throw; BPU—ballistic push-up; CG—control group; CMJ—countermovement jump; CS—cluster set; D—discipline; DBPT—dumbbell press test; E—elite; ERP—elastic resistance punch; F—female; GPT—general performance test; HPT—hip power test; HRE—heavy resistance exercise; HS—half-squat; ISO—isokinetic test; ISO EXT—isokinetic extensions; ISOP—isometric punch; ISR—intra-set rest; J—jump; JU—judo; KAR—karate; KBOX—kickboxing; L—load as SETSxREPS@INTENSITY; LSJ—loaded squat jump; LVL—competitive level; M—mean; M—male; MIX—mixed; MT—Muay Thai; *n*—sample size; NR—not reported; P—plyometrics; PBPT—power bench press test; RAST—running anaerobic sprint test; REF—reference; RHIT—repeated high-intensity techniques; RI—rest intervals; SD—standard deviation; SQ—squat; SSR—self-selected rest interval; TJ—tuck jump; TKD—taekwondo; TS—traditional set; TW—taolo wushu; WBV—whole-body vibration; WRE—wrestling; X—lack of data; Y—yes.

**Table 4:** Conditioning activities of studies included in the meta-analysis.

Author	#	Conditioning Activity/Discipline Competitive Level
Yi et al., 2022 [32]	CA1	Loaded squat jumps 4 × 8 × 30%1RM, 3 min rest, 90 s, intraset rest/B-A
	CA2	Loaded squat jumps 4 × 8 × 30%1RM, 6 min rest, 90 s, intraset rest/B-A
	CA3	Loaded squat jumps 4 × 8 × 30%1RM, 9 min rest, 90 s, intraset rest/B-A
	CA4	Loaded squat jumps 4 × 8 × 30%1RM, 12 min rest, 90 s, intraset rest/B-A
	CA5	Squat 3 × 5 × 80%1RM, 3 min rest, 9 -s, intraset rest/B-A
	CA6	Squat 3 × 5 × 80%1RM, 6 min rest, 90 s, intraset rest/B-A
	CA7	Squat 3 × 5 × 80%1RM, 9 min rest, 90 s, intraset rest/B-A
	CA8	Squat 3 × 5 × 80%1RM, 12 min rest, 90 s, intraset rest/B-A
Ouergui et al., 2022 [25]	CA9	Bandal chagui, 3 × 5 s, 10 min rest, 30 s intraset rest/T-A
	CA10	Bandal chagui, 3 × 5 s, 10 min rest, 35 s intraset rest/T-A
	CA11	Bandal chagui, 3 × 5 s, 10 min rest, self-selected intraset rest/T-A
	CA12	Consutive vertical jump, 3 × 5 s, 10 min rest, 30 s intraset rest/T-A
	CA13	Consecutive vertical jump, 3 × 5 s, 10 min rest, 35 s intraset rest/T-A
	CA14	Consecutive vertical jump, 3 × 5 s, 10 min rest, self-selected interset rest/T-A
Margaritopoulos, et al., 2015 [37]	CA15	Tuck jumps, 3 × 5, 5 min rest, intraset rest 30 sek (a)/K-A
	CA16	Tuck jumps, 3 × 5, 5 min rest, intraset rest 30 sek (b)/K-A
	CA17	Tuck jumps, 3 × 5, 5 min rest, intraset rest 30 sek (c)/K-A
	CA18	Control, 5 min rest, (a)/K-A
	CA19	Control, 5 min rest, (b)/K-A
	CA20	Control, 5 min rest, (c)/K-A
Da Silva Santos, et al., 2015 [26]	CA21	Half squat, 3 × 1 × 95%1RM, 5 min rest, 3 min interset rest/T-E
	CA22	Half squat, 3 × 1 × 95%1RM, 10 min rest, 3 min interset rest/T-E
	CA23	Half squat, 3 × 1 × 95%1RM, self-selected rest, 3 min interset rest/T-E
	CA24	Jumps, 3 × 10, 5 min rest, 30 s interset rest/T-E
	CA25	Jumps, 3 × 10, 10 min rest, 30 s interset rest/T-E
	CA26	Jumps, 3 × 10,self-selected rest, 30 s interset rest/T-E
	CA27	Half squat + jumps, 3 × 2 × 95%1RM + 4, 5 min rest, 3 min interset rest/T-E
	CA28	Half squat + jumps, 3 × 2 × 95%1RM + 4, 10 min rest, 3 min interset rest/T-E
	CA29	Half squat + jumps, 3 × 2 × 95%1RM + 4, self-selected rest, 3 min interset rest/T-E
Da Silva Santos, et al., 2016 [12]	CA30	Half-squat, 1 × 3 × 50%1RM, 10 min rest/T-E
	CA31	Half-squat, 1 × 3 × 90%1RM, 10 min rest/T-E
	CA32	Half-squat, 3 × 3 × 50%1RM, 10 min rest/T-E
	CA33	Half-squat, 3 × 3 × 90%1RM, 10 min rest/T-E
Cimadoro, et al., 2018 [39]	CA34	Roundhouse kicks, 20 × 1, 0 min rest, 1 s interset rest/M-A
	CA35	Roundhouse kicks, 20 × 1, 5 min rest, 1 s interset rest/M-A
	CA36	Roundhouse kicks, 20 × 1, 10 min rest, 1 s interset rest/M-A
	CA37	Roundhouse kicks, 20 × 1, 20 min rest, 1 s interset rest/M-A
	CA38	Roundhouse kicks, 20 × 1, 30 min rest, 1 s interset rest/M-A
	CA39	Roundhouse kicks, 20 × 1, 0 min rest, 3 s interset rest/M-A
	CA40	Roundhouse kicks, 20 × 1, 5 min rest, 3 s interset rest/M-A
	CA41	Roundhouse kicks, 20 × 1, 10 min rest, 3 s interset rest/M-A
	CA42	Roundhouse kicks, 20 × 1, 20 min rest, 3 s interset rest/M-A
	CA43	Roundhouse kicks, 20 × 1, 30 min rest, 3 s interset rest/M-A
Oliviera, et al., 2018 [29]	CA44	Whole-body vibration 1 × 1 min x 26 hz/T-A
Ouergui et al., 2023 [30]	CA45	Bandal chagui, 3 × 5 s, 3 min rest, 30 s intraset rest/T-A
	CA46	Bandal chagui, 3 × 5 s, 3 min rest, 45 s intraset rest/T-A
	CA47	Bandal chagui, 3 × 5 s, 3 min rest, self-selected intraset rest/T-A
	CA48	Consecutive vertcial jump, 3 × 5 s, 3 min rest, 30 s intraset rest/T-A
	CA49	Consecutive vertcial jump, 3 × 5 s, 3 min rest, 45 s intraset rest/T-A
	CA50	Consecutive vertcial jump, 3 × 5 s, 3 min rest, self-selected interset rest/T-A
	CA51	Bandal chagui, 3 × 5 s, 7 min rest, 30 s intraset rest/T-A
	CA52	Bandal chagui, 3 × 5 s, 7 min rest, 45 s intraset rest/T-A
	CA53	Bandal chagui, 3 × 5 s, 7 min rest, self-selected intraset rest/T-A
	CA54	Consecutive vertical jump, 3 × 5 s, 7 min rest, 30 s intraset rest/T-A
	CA55	Consecutive vertical jump, 3 × 5 s, 7 min rest, 45 s intraset rest/T-A
	CA56	Consecutive vertical jump, 3 × 5 s, 7 min rest, self-selected interset rest/T-A
Boyaci and Kizilet., 2023 [31]	CA57	Squat, 3 × 12 × 75%1RM, 30 s rest, 180 s interset/T-E
	CA58	Squat, 3 × 12 × 75%1RM, 4-min rest, 180 s interset/T-E
	CA59	Squat, 3 × 12 × 75%1RM, 8-min rest, 180 s interset/T-E
	CA60	Squat, 3 × (4 + 4 + 4) × 75%1RM, 30-s rest, 180 s interset/T-E
	CA61	Squat, 3 × (4 + 4 + 4) × 75%1RM, 4 min rest, 180 s interset/T-E
	CA62	Squat, 3 × (4 + 4 + 4) × 75%1RM, 8 min rest, 180 s interset/T-E

1RM—one maximal repetition test; combat sport: B—boxing, K—karate, M—Muay Thai, T—taekwondo; competition level: A—amateur, E—elite; CA—conditioning activity.

**Table 5:** Overview of the meta-analysis encompassing all disciplines.

Name	Sample Size	ES	SE	±95%CI	Z-Statistic	*p*-Palue	Variance	Weight
CA1	10	−0.2	0.45	−1.08; 0.68	−0.44	0.66	0.20	3.28
CA2	10	0	0.45	−0.88; 0.88	0.00	1.00	0.20	3.28
CA3	10	0.03	0.45	−0.85; 0.91	0.07	0.95	0.20	3.28
CA4	10	−0.02	0.45	−0.90; 0.86	−0.04	0.96	0.20	3.28
CA5	10	−0.02	0.45	−0.90; 0.86	−0.04	0.96	0.20	3.28
CA6	10	−0.06	0.45	−0.94; 0.82	−0.13	0.89	0.20	3.28
CA7	10	0.03	0.45	−0.85; 0.91	0.07	0.95	0.20	3.28
CA8	10	−0.04	0.45	−0.92; 0.84	−0.09	0.93	0.20	3.28
CA9	27	0.09	0.27	−0.44; 0.84	0.33	0.74	0.07	5.70
CA10	27	0.09	0.27	−0.44; 0.62	0.33	0.74	0.07	5.70
CA11	27	0.14	0.27	−0.39; 0.67	0.52	0.60	0.07	5.70
CA12	27	−0.21	0.27	−0.74; 0.32	−0.78	0.44	0.07	5.70
CA13	27	0.23	0.27	−0.30; 0.76	0.85	0.39	0.07	5.70
CA14	27	−0.04	0.27	−0.57; 0.49	−0.15	0.88	0.07	5.70
CA15	10	0.17	0.45	−0.71; 1.05	0.38	0.71	0.20	3.28
CA16	10	0	0.45	−0.88; 0.88	0.00	1.00	0.20	3.28
CA17	10	0.14	0.45	−0.74; 1.02	0.31	0.76	0.20	3.28
CA18	10	−0.2	0.45	−1.08; 0.68	−0.44	0.66	0.20	3.28
CA19	10	−0.22	0.45	−1.10; 0.66	−0.49	0.62	0.20	3.28
CA20	10	−0.37	0.45	−1.25; 0.51	−0.82	0.41	0.20	3.28
CA21	11	−0.06	0.43	−0.90; 0.78	−0.14	0.89	0.18	3.48
CA22	11	0	0.43	−0.84; 0.84	0.00	1.00	0.18	3.48
CA23	11	−0.11	0.43	−0.95; 0.73	−0.26	0.80	0.18	3.48
CA24	11	−0.17	0.43	−1.01; 0.67	−0.40	0.69	0.18	3.48
CA25	11	−0.16	0.43	−1.00; 0.68	−0.37	0.71	0.18	3.48
CA26	11	0.19	0.43	−0.65; 1.03	0.44	0.66	0.18	3.48
CA27	11	0.04	0.43	−0.80; 0.88	0.09	0.93	0.18	3.48
CA28	11	0.17	0.43	−0.67; 1.01	0.40	0.69	0.18	3.48
CA29	11	0.19	0.43	−0.65; 1.03	0.44	0.66	0.18	3.48
CA30	9	−0.19	0.47	−1.11; 0.73	−0.40	0.69	0.22	3.09
CA31	9	0.08	0.47	−0.84; 1.00	0.17	0.86	0.22	3.09
CA32	9	−0.31	0.47	−1.23; 0.61	−0.66	0.51	0.22	3.09
CA33	9	0.17	0.47	−0.75; 1.09	0.36	0.72	0.22	3.09
CA34	9	−0.53	0.48	−1.47; 0.41	−1.10	0.27	0.23	3.00
CA35	9	−0.24	0.47	−1.16; 0.68	−0.51	0.61	0.22	3.09
CA36	9	−0.37	0.48	−1.31; 0.57	−0.77	0.44	0.23	3.00
CA37	9	−0.95	0.5	−1.93; 0.03	−1.90	0.06	0.25	2.84
CA38	9	−1.36	0.52	−2.38; −0.34	−2.62	0.009 *	0.27	2.68
CA39	9	−0.37	0.48	−1.31; 0.57	−0.77	0.44	0.23	3.00
CA40	9	−0.31	0.47	−1.23; 0.61	−0.66	0.51	0.22	3.09
CA41	9	−0.71	0.49	−1.67; 0.25	−1.45	0.15	0.24	2.92
CA42	9	−1.14	0.51	−2.14; −0.14	−2.24	0.025 *	0.26	2.76
CA43	9	−1.33	0.52	−2.35; −0.31	−2.56	0.011 *	0.27	2.68
CA44	15	0.02	0.37	−0.71; 0.75	0.05	0.96	0.14	4.18
CA45	21	0.23	0.31	−0.38; 0.84	0.74	0.46	0.10	5.04
CA46	21	0.66	0.32	0.03; 1.29	2.06	0.039 *	0.10	4.88
CA47	21	0.84	0.32	0.21; 1.47	2.63	0.009 *	0.10	4.88
CA48	21	0.53	0.31	−0.08; 1.14	1.71	0.09	0.10	5.04
CA49	21	0.93	0.32	0.30; 1.56	2.91	0.004 *	0.10	4.88
CA50	21	0.95	0.33	0.30; 1.60	2.88	0.004 *	0.11	4.73
CA51	21	1.19	0.33	0.54; 1.84	3.61	0.000 *	0.11	4.73
CA52	21	0.93	0.32	0.30; 1.56	2.91	0.004 *	0.10	4.88
CA53	21	0.81	0.32	0.18; 1.44	2.53	0.011 *	0.10	4.88
CA54	21	0.82	0.32	0.19; 1.45	2.56	0.010 *	0.10	4.88
CA55	21	0.65	0.32	0.02; 1.28	2.03	0.042 *	0.10	4.88
CA56	21	0.7	0.32	0.07; 1.33	2.19	0.029 *	0.10	4.88
CA57	12	0.04	0.41	−0.76; 0.84	0.10	0.92	0.17	3.70
CA58	12	0.38	0.41	−0.42; 1.18	0.93	0.35	0.17	3.70
CA59	12	0.21	0.41	−0.59; 1.01	0.51	0.61	0.17	3.70
CA60	12	0.53	0.42	−0.29; 1.35	1.26	0.21	0.18	3.59
CA61	12	1.07	0.44	0.21; 1.93	2.43	0.015 *	0.19	3.38
CA62	12	0.34	0.41	−0.46; 1.14	0.83	0.41	0.17	3.70
Total	866	0.136	0.065	0.008; 0.263	2.090	0.037 *		

CA—conditioning activity; ES—effect size; SE—standard error; ±95%CI—confidence interval (lower; upper); *—statistical significance.

**Table 6:** Overview of the meta-analysis by discipline—aggregated view.

Name	Sample Size	ES	SE	±95%CI	Z-Statistic	*p*-Value	Variance	Weight
Boxing	80	−0.035	0.159	−0.347; 0.277	−0.220	0.826	0.025	39.506
Karate	60	−0.080	0.184	−0.440; 0.280	−0.435	0.663	0.034	29.630
Muay Thai	90	−0.701	0.155	−1.006; −0.397	−4.517	0.00001 *	0.024	41.481
Taekwondo	636	0.346	0.067	0.214; 0.477	5.137	<0.000001 *	0.005	220.925
Total	866	0.131	0.055	0.024; 0.239	2.389	0.017 *		

CA—conditioning activity; ES—effect size; SE—standard error; ±95%CI—confidence interval (lower; upper); *—statistical significance.

**Table 7:** Overview of the meta-analysis by competitive level—aggregated view.

Name	Sample Size	ES	SE	±95%CI	Z-Statistic	*p*-Value	Variance	Weight	Contribution
Amateur	659	0.14	0.08	−0.01; 0.29	1.77	0.08	0.01	169.53	0.72
Elite	207	0.13	0.12	−0.11; 0.38	1.06	0.29	0.02	64.69	0.28
Total	866	0.13	0.07	0.01; 0.26	2.06	0.039 *			

CA—conditioning activity; SE—standard error; ±95%CI—confidence interval (lower; upper); *—statistical significance.

## Data Availability

The data supporting the findings of this study are available upon reasonable request from the corresponding author.

## Abbreviations

The following abbreviations are used in this manuscript:

PAPE	Post-activation performance enhancement
PAP	Post-activation potentiation
CMJ	Countermovement jump
MVIC	Maximum voluntary isometric contraction
MRIC	Myosin regulatory light chain
RCT	Randomized controlled trial
PEDro	Physiotherapy Evidence Database
1RM	One-repetition maximum
MMA	Mixed martial arts
RFD	Rate of force development
LSJ	Loaded squat jump
BP	Bench press
BPT	Bench press throw
BPU	Ballistic push-up
HS	Half-squat
ISOP	Isometric punch
ISR	Intra-set rest
SSR	Self-selected rest
TW	Taolu wushu
HRE	Heavy-resistance exercise
HPT	Hip power test
PBPT	Power bench press test
RAST	Running anaerobic sprint test
DBPT	Dumbbell press test
